# 微生物宏蛋白质组——从样品处理、数据采集到数据分析

**DOI:** 10.3724/SP.J.1123.2024.02009

**Published:** 2024-07-08

**Authors:** Enhui WU, Liang QIAO

**Affiliations:** 复旦大学化学系,上海 200433; Department of Chemistry, Fudan University, Shanghai 200433, China

**Keywords:** 宏蛋白质组学, 样品前处理, 数据库, 数据分析策略, metaproteomics, sample pretreatment, database, data analysis strategy

## Abstract

微生物与人体疾病、健康密切相关,如何理解微生物群落的组成及其发挥的功能是一大亟需研究的问题。近年来,宏蛋白质组学已经成为研究微生物组成与功能的重要技术手段。然而,由于微生物群落样本的复杂性与高度异质性,样品处理、质谱数据采集与数据分析成为宏蛋白质组目前面临的三大挑战。在宏蛋白质组分析中往往需要针对不同类型的样品进行前处理优化,采取不同的微生物分离富集、提取和裂解方案。与单一物种蛋白质组相类似,宏蛋白质组学中的质谱数据采集模式有数据依赖性采集(data-dependent acquisition, DDA)模式和数据非依赖性采集(data-independent acquisition, DIA)模式。DIA数据采集模式可以完整地采集样品的肽段信息,具有很强的发展潜力。但是由于宏蛋白质组样品的复杂性,其DIA数据解析已成为阻碍宏蛋白质组深度覆盖的一大难题。在数据解析方面,最重要的步骤在于蛋白质序列数据库的构建。数据库的大小和完整性不仅对鉴定数量有很大影响,还会影响物种和功能水平上的分析。目前宏蛋白质组数据库构建的金标准是基于宏基因组的蛋白质序列数据库。同时,基于迭代搜库的公共数据库过滤方法也已被证明具有很强的实用价值。从具体的数据解析策略角度,以肽段为中心的DIA数据解析方法占据了绝对的主流。随着深度学习和人工智能的发展,其会极大地推动宏蛋白质组数据解析的准确度、覆盖度与分析速度。在下游生物信息学分析方面,近年来开发了一系列注释工具,可以在蛋白水平、肽段水平、基因水平上进行物种注释来获得微生物群落组成。与其他组学方法相比,微生物群落的功能分析是宏蛋白质组学的一个独特特征。宏蛋白质组已经成为微生物群落多组学分析中的重要组成部分,并且仍在覆盖深度、检测灵敏度、数据解析完整度等方面具有很大的发展潜力。

## Abstract (en)

Microorganisms are closely associated with human diseases and health. Understanding the composition and function of microbial communities requires extensive research. Metaproteomics has recently become an important method for throughout and in-depth study of microorganisms. However, major challenges in terms of sample processing, mass spectrometric data acquisition, and data analysis limit the development of metaproteomics owing to the complexity and high heterogeneity of microbial community samples. In metaproteomic analysis, optimizing the preprocessing method for different types of samples and adopting different microbial isolation, enrichment, extraction, and lysis schemes are often necessary. Similar to those for single-species proteomics, the mass spectrometric data acquisition modes for metaproteomics include data-dependent acquisition (DDA) and data-independent acquisition (DIA). DIA can collect comprehensive peptide information from a sample and holds great potential for future development. However, data analysis for DIA is challenged by the complexity of metaproteome samples, which hinders the deeper coverage of metaproteomes. The most important step in data analysis is the construction of a protein sequence database. The size and completeness of the database strongly influence not only the number of identifications, but also analyses at the species and functional levels. The current gold standard for metaproteome database construction is the metagenomic sequencing-based protein sequence database. A public database-filtering method based on an iterative database search has been proven to have strong practical value. The peptide-centric DIA data analysis method is a mainstream data analysis strategy. The development of deep learning and artificial intelligence will greatly promote the accuracy, coverage, and speed of metaproteomic analysis. In terms of downstream bioinformatics analysis, a series of annotation tools that can perform species annotation at the protein, peptide, and gene levels has been developed in recent years to determine the composition of microbial communities. The functional analysis of microbial communities is a unique feature of metaproteomics compared with other omics approaches. Metaproteomics has become an important component of the multi-omics analysis of microbial communities, and has great development potential in terms of depth of coverage, sensitivity of detection, and completeness of data analysis.

微生物群落在地球生态系统中扮演着至关重要的角色,包括人类在内的几乎所有的生命体都与周围的微生物密切相关。人体内栖居着海量的微生物,包括细菌、古细菌、病毒和单细胞真核生物等。这些微生物可以通过与宿主之间的相互作用执行复杂的功能,对人体的疾病和健康产生深远的影响。大量研究证明人体微生物与癌症、糖尿病、神经系统疾病等疾病之间存在关联关系^[1⇓⇓-4]^。因此,全面深入地了解微生物群落的结构和功能对于维持人体健康至关重要。在微生物组研究中,16S核糖体RNA (16S rRNA)测序是目前研究微生物群落组成最常用的方法^[5]^。然而这种方法只能评估微生物群落的多样性和群落组成,不能分析微生物群落的实际功能。为了真实地检测微生物群落的功能潜力,研究人员使用高通量测序技术(NGS)来分析微生物群落的整个宏基因组^[6,7]^。随着测序技术的发展、测序通量的提高和测序成本的下降,宏基因组在微生物群落研究中的应用越来越多,极大地推动了微生物组学的进展。蛋白质作为生命活动的重要参与者,直接参与多种细胞生理过程,如激素调节、基因表达、细胞间通讯等。虽然宏基因组学可以通过分析微生物群落的全部基因揭示特定群落的功能潜力,但直接检测蛋白质对于理解微生物群落实际表达的功能更为重要。宏蛋白质组通过全面检测微生物群落所表达的蛋白质,可以提供关于微生物群落结构与功能的深入视角。

2004年,Wilmes等^[8]^首次提出了宏蛋白质组的概念,即特定时刻下微生物群落表达的全部蛋白质,并将其应用在活性污泥微生物的研究中,开创了使用宏蛋白质组研究微生物群落的先河。迄今为止,宏蛋白质组已经经过了逾20年的发展历程,其在微生物组学研究领域的应用变得越来越广泛,已经成为研究微生物群落功能的一项强大而有效的技术手段^[9⇓⇓-12]^。为了更直观地理解这一领域的发展速度,我们采用“metaproteomics”作为关键词,在PubMed上检索并绘制了过去20年的相关文献数量变化图(图1),并简要绘制了宏蛋白质组学发展过程中几个里程碑式的进展及其时间节点(图2)。可以看到,近年来发表的关于宏蛋白质组的文章数量开始迅速增长,宏蛋白质组的应用范围也在不断拓宽。

**图1:**
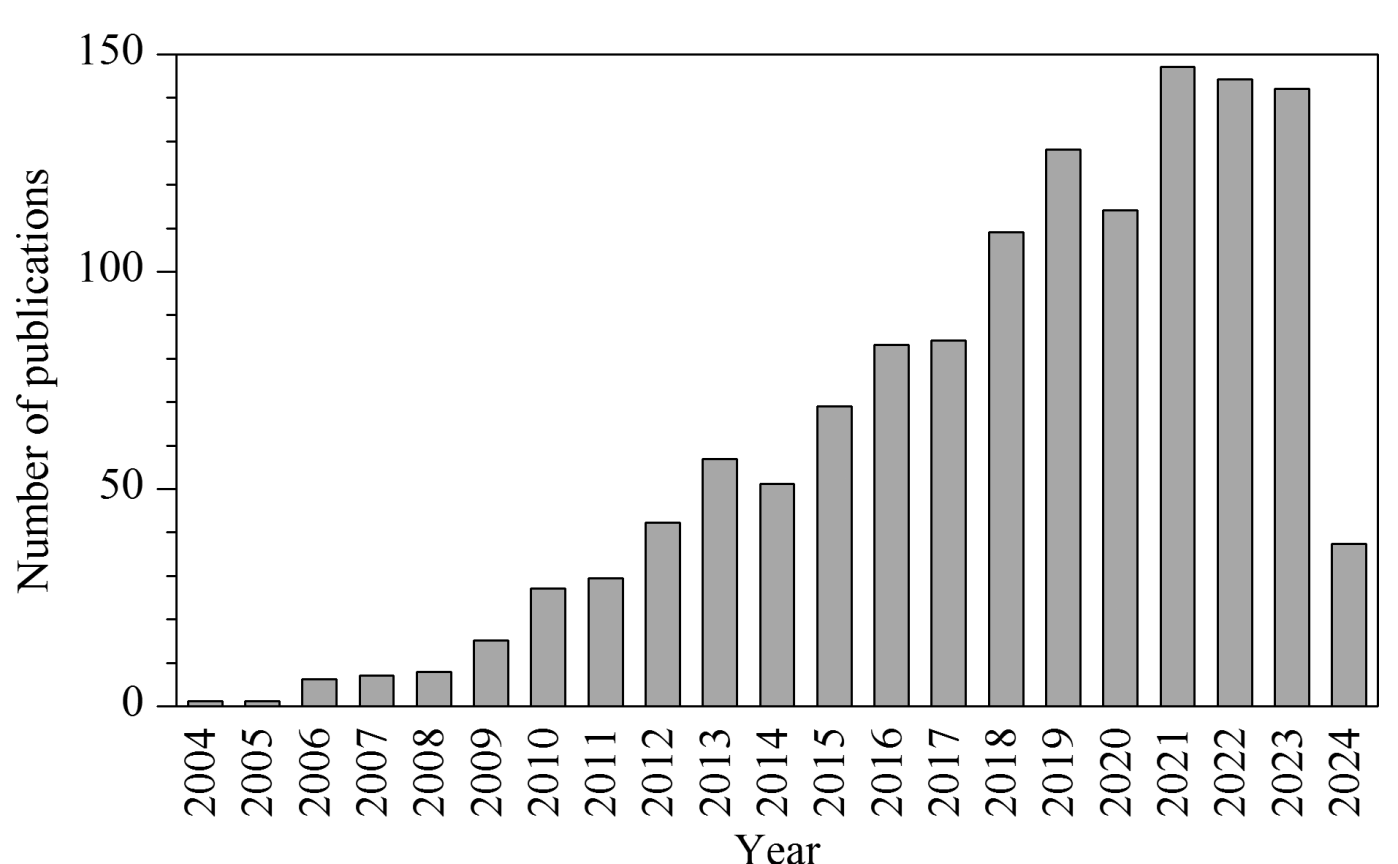
宏蛋白质组相关文章的发表数量变化

**图2:**
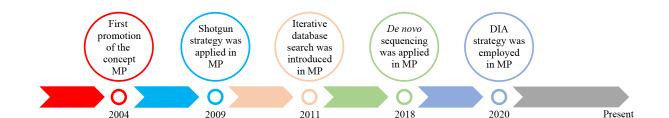
宏蛋白质组技术里程碑式进展时间图

最初几年的宏蛋白质组研究主要依赖于二维凝胶电泳分离蛋白质,然后采用四极杆-飞行时间质谱(Q-TOF)、基质辅助激光解吸飞行时间质谱(MALDI-TOF)等质谱仪器对单个蛋白质点进行分析^[8,13⇓-15]^。然而,这种方法通量非常低,且非常耗时。2009年,Verberkmoes等^[16]^首次采用基于线性离子阱轨道阱组合式质谱(LTQ Orbitrap)的鸟枪法宏蛋白质组分析人体粪便样本,实现了基于液相色谱-串联质谱(LC-MS/MS)的微生物蛋白质的大规模表征。随后,为了提高宏蛋白质组分析的准确性和覆盖深度,研究者们开发了各种数据分析方法,比如迭代搜库^[17]^和从头测序(*de novo* sequencing)策略^[18,19]^来优化数据依赖性采集(data-dependent acquisition, DDA)宏蛋白质组分析。近几年来,随着高分辨率质谱仪的发展以及新型质谱采集策略如数据非依赖性采集策略(data-independent acquisition, DIA)开始被应用于宏蛋白质组^[20,21]^,宏蛋白质组学分析在深度和准确度方面得到了显著的提升,为复杂生物样本的蛋白质组分析提供了更高的解析能力和更广泛的应用前景。

当前,宏蛋白质组的分析主要采用基于LC-MS/MS的鸟枪法策略,其基本流程如图3所示:首先进行样本前处理,将微生物尽可能从复杂成分中分离出来。然后采用合适的裂解方式提取微生物蛋白,再将蛋白质混合物酶解成肽段混合物后,对肽段混合物进行色谱分离和质谱数据采集。根据获得的质谱数据信息进行肽段鉴定和蛋白质推断,获得样本的蛋白质鉴定和定量结果。最后进行物种分类和功能注释,挖掘具有重要生物学意义的功能蛋白信息。然而,与传统的单一物种蛋白质组学不同,宏蛋白质组的实际样本中可能存在上百种不同微生物,具有高度复杂性和分类多样性,因此给宏蛋白质组分析带来了许多挑战。本文将基于当前宏蛋白质组数据分析策略和技术难点,介绍宏蛋白质组的研究方法和研究现状。

**图3:**
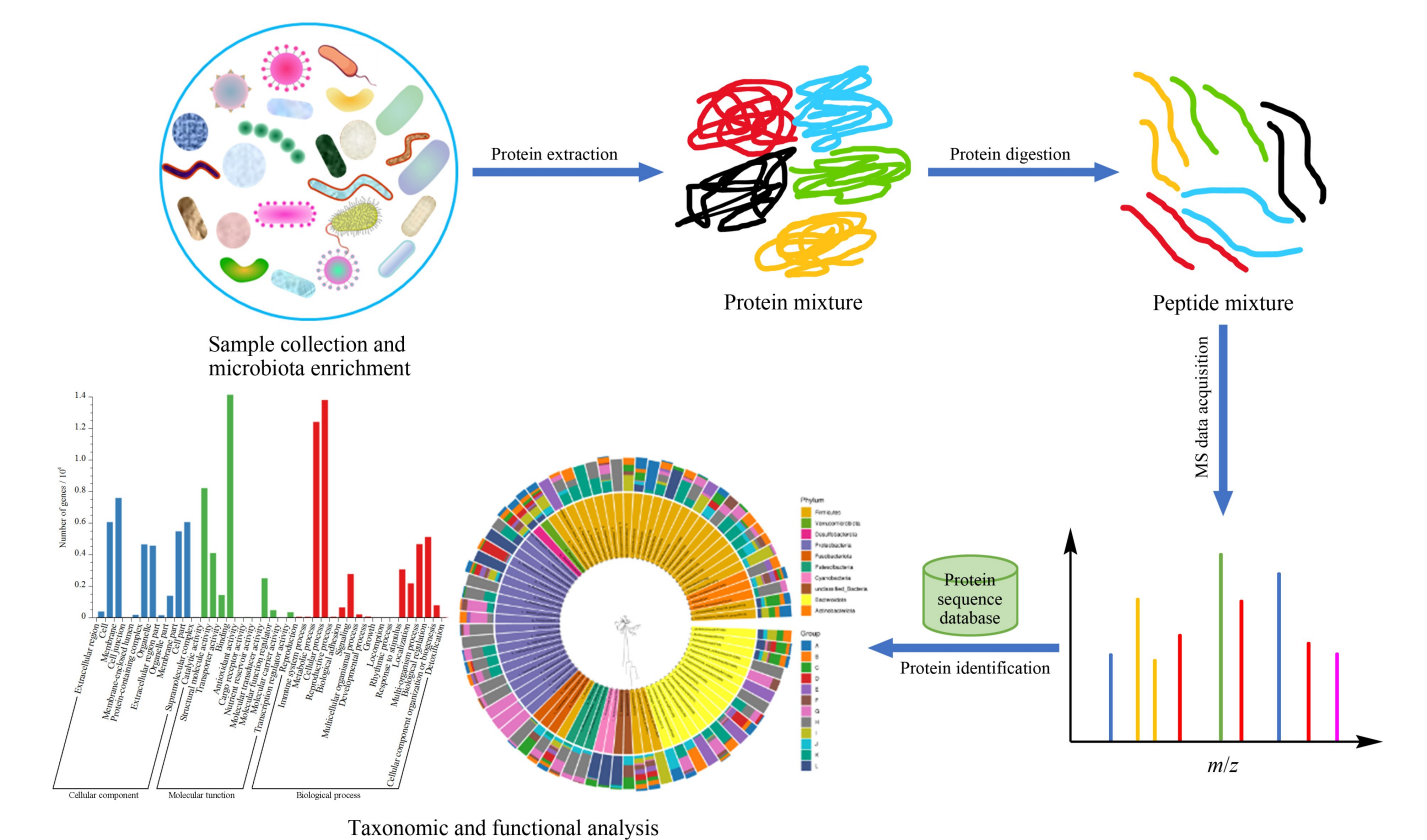
鸟枪法宏蛋白质组分析流程

## 1 样品前处理

目前,宏蛋白质组学技术已经被广泛应用于人体微生物组、土壤、食品、海洋、活性淤泥等多个领域的研究^[22⇓⇓⇓-26]^。与单一物种的蛋白质组分析相比,复杂样本的宏蛋白质组样品前处理面临更多挑战。实际样本中微生物组成复杂,丰度动态范围大,不同类型的微生物细胞壁结构存在很大差异,而且样本中往往包含大量宿主蛋白以及其他杂质的背景干扰。因此,在宏蛋白质组分析中往往需要针对不同类型的样品进行优化,采取不同的微生物分离富集、提取和裂解方案。

肠道菌群是人体微生物宏蛋白质组研究中最热门的领域,而粪便样本因其易获取性和代表性在肠道菌群研究中被广泛使用。粪便样本中包含了微生物、宿主细胞/蛋白质和食物残渣等混合物,需要采用差速离心或滤膜过滤等方法进行微生物的分离和富集^[27⇓-29]^。常用的从粪便样本微生物中提取蛋白质的方法主要包括超声破碎法、珠磨破碎法、液氮研磨法、十二烷基磺酸钠(sodium dodecyl sulfonate, SDS)裂解等化学裂解法以及他们的组合等方法^[30⇓-32]^。口腔微生物组作为人体中第二大的微生物群落,也受到广泛的关注,差速离心与过滤法等用于粪便样品的前处理策略也被应用在口腔微生物组的研究中。最近,Jiang等^[33]^将高分辨自由流等电聚焦电泳技术应用于口腔微生物分离中,实现了口腔微生物蛋白的深度鉴定(鉴定到3647种蛋白质),且使肽段和物种的鉴定数量分别增加了94.97%和44.90%。利用该方法,研究人员^[33]^对口腔微生物的物种组成和蛋白质功能进行了探究,并系统比较了肺癌患者口腔微生物组与正常对照组的差异,发现并验证了两种在肺癌患者中功能表达紊乱的口腔微生物。

在土壤宏蛋白质组的研究中,由于土壤具有复杂的物理结构、异质性和有机化合物的干扰,其前处理的一大关键点在于将蛋白质从腐殖质等复杂有机化合物中分离出来^[34]^。SDS-酚、SDS-三氯乙酸(trichloroacetic acid, TCA)裂解与滤膜过滤相结合、基于NaOH预处理等方法被用于提取土壤微生物蛋白质^[34⇓-36]^。此外,商业化试剂盒NoviPure Soil Protein Kit也被用于土壤宏蛋白质组的研究,取得了与传统方法相当甚至更好的结果^[34,37]^。活性淤泥成分非常复杂,通常由细菌、原生生物、真菌等微生物群体形成的颗粒或絮体组成,是污水处理过程中的关键生物组成部分。珠磨破碎、超声破碎、SDS裂解等方法也被应用于活性污泥样品的微生物蛋白质提取^[26,38]^。最近的一项研究中,Kleikamp等^[39]^将珠磨破碎和冻融法应用于污水处理厂的好氧颗粒污泥样品前处理中,并首次采用宏蛋白质组、宏基因组和16S rRNA测序技术进行微生物群落的多组学对比研究,结果表明不同组学技术在低分类水平上的分类组成具有显著差异。海洋微生物主要包括浮游生活的微生物和附着在颗粒物上生活的微生物。Schultz等^[40]^比较了6种蛋白质提取方案,包括(1)苯酚提取、(2)SDS-TCA裂解、(3)SDS-丙酮裂解、(4)珠磨破碎法、(5)冻融法以及(6)商业化试剂盒TRI-Reagent,最终证明使用SDS-丙酮裂解或者珠磨破碎的方法能获得较高的蛋白产率。

传统发酵食品和饮料的化学环境差异大,含有多种组成不同的微生物,如细菌、真菌、酵母等^[22]^。Zhao等^[41]^比较了不同细胞破碎方法(组织研磨仪研磨和液氮研磨)、不同裂解液(SDS和硼砂/聚乙烯吡咯烷酮/苯酚(borax/polyvinylpolypyrrolidone/phenol, BPP))以及不同肽段纯化方法(C_18_柱、固相烷基化试剂、肽段纯化试剂),在高温大曲样品中的应用结果,证明采用液氮研磨进行细胞破碎后,用BPP裂解液提取蛋白质,然后用C_18_柱对酶解后的肽段进行纯化可以取得优于其他方法的结果。利用该方法处理黄曲样品,可以从中鉴定到3350个蛋白质和10202条特征肽段,而通过其他方法最多只能鉴定到2029个蛋白质和3477条肽段。应用优化的宏蛋白组学方法,作者分析了在春季、夏季和秋季收集的90个白曲、黄曲和黑曲样品,揭示了黄曲具有更高丰度的糖化酶,有利于原料的降解,在黑曲中发现微生物蛋白丰度随季节显著变化,证明了秋季黑曲碳水化合物和氨基酸代谢相关通路蛋白质表达量升高。此外,作者通过宏蛋白质组对一年中6个生产周期的42份高温大曲样本进行了差异蛋白质功能和代谢通路分析,揭示了大曲代谢途径与白酒发酵过程的相关性,从而形成中国白酒特有的风味和香气^[42]^。

综上所述,不同样品中的微生物宏蛋白质组提取具有一定的相似性也具有部分差异性,但是目前对于不同种类的宏蛋白质组样品均缺乏一个统一的前处理流程。

## 2 质谱数据采集

鸟枪法宏蛋白质组分析中,预处理之后的肽段混合物首先在色谱柱中分离,然后进入质谱仪经离子化后进行数据采集。与单一物种蛋白质组分析相类似,宏蛋白质组分析中的质谱数据采集模式有DDA模式和DIA模式。在DDA模式中,肽段离子在进行一级质谱(MS^1^)扫描后,采集MS^1^中信号强度最高的多个母离子分别进行二级质谱(MS^2^)碎裂和采集。在DDA数据采集模式中,得到的每张MS^2^谱图都可以与单一母离子对应,因此可以根据MS^2^信息进行肽段定性分析。但是这种采集方式是丰度依赖性的,在低丰度肽段信息采集上有较强的随机性,限制了其检测灵敏度,并且会导致较多的缺失值和较低的重复性,因此不适合大队列样本的定量分析。尤其是对于复杂的宏蛋白质组样品来说,微生物群落的物种丰度往往呈现较宽的动态范围,导致其蛋白质和肽段层面的丰度差异巨大。那些低丰度的肽段往往在MS^1^中具有较低的信号强度,因此很难被选中进行MS^2^分析。为了克服DDA技术的局限性,研究人员开发了DIA数据采集模式^[43,44]^。在DIA模式中,MS^1^扫描区间依据其质荷比被分为若干个隔离窗口,每个窗口的所有离子共同碎裂和检测,最终得到包含全部碎片离子信息的MS^2^谱图。该方法具有系统性、无偏向性采集的特点,样本缺失值少,重现性和稳定性高,因此可以实现大队列样本分析。

随着质谱仪器的不断迭代更新,更高灵敏度和分辨率的质谱仪器被应用于宏蛋白质组中,宏蛋白质组分析的覆盖深度也因而不断提升。长期以来,以Orbitrap为首的一系列高分辨率质谱仪器在宏蛋白质组中被广泛应用。近几年来,Bruker公司开发的一系列捕集离子淌度(trapped ion mobility spectrometry, TIMS)质谱,如timsTOF Pro 2、timsTOF HT和timsTOF Ultra,采用双TIMS技术和同步累积连续碎裂(parallel accumulation-serial fragmentation, PASEF)数据采集模式,实现了蛋白质组分析更高的覆盖度。TIMS PASEF扫描模式可以达到近100%的离子利用率,实现极高的灵敏度和扫描速度,使蛋白质组分析在鉴定深度和精度、定量准确性、动态范围、灵敏度等方面有了全面提升。TIMS-PASEF技术已经在蛋白质组学研究中广泛应用^[45,46]^,在宏蛋白质组学领域也表现出很大的应用潜力,未来在临床大队列微量样本的宏蛋白质组学分析中具有广阔的应用前景。Gómez-Varela等^[47]^使用timsTOF Pro进行DDA-PASEF和DIA-PASEF的宏蛋白质组分析。结果表明,与之前发表的DDA和DIA方法相比,使用DDA-PASEF可将肽段定量能力提高5倍,并具有更高的准确性和重复性。此外,DIA-PASEF扩大了检测到的低丰度蛋白质的动态范围,应用该技术使未知功能蛋白质的定量数目翻倍。与之前的DDA宏蛋白质组分析相比,DIA-PASEF的质谱采集时间缩短为原来的1/16,色谱梯度时间缩短为原来的1/4,量化出高达4倍的分类单元。在小鼠模型上的应用揭示了疼痛相关的蛋白质复合物的动态变化,这些复合物在宿主免疫系统与肠道微生物之间的信息交流中起关键作用。

表1从样品类型、分析策略、质谱仪器、采集方法、分析软件、鉴定数目等层面展示了2011年至今宏蛋白质组学的部分代表性研究。

**表1:** 2011年至今宏蛋白质组学的部分代表性研究

Reference	Time	Samples	Analysis strategy	MS instrument	MS acquisition mode	Analysis tools	Identification
Rooijers et al.^[17]^	2011	fecal sample	iterative search	LTQ-Orbitrap	DDA	OMSSA	over 3000 peptides
Jagtap et al.^[48]^	2012	salivary sample	iterative search	LTQ-Orbitrap	DDA	MaxQuant	2176 proteins
Tanca et al.^[49]^	2013	synthetic microbial community	metagenomics derived databases	LTQ-Orbitrap	DDA	Proteome Discoverer	10881 peptides
Zhang et al.^[50]^	2016	mouse fecal sample; human colonoscopy sample	MetaPro-IQ	Q Exactive	DDA	X! Tandem MaxQuant	30749 proteins; 19011 proteins
May et al.^[51]^	2016	ocean microbiome sample	Metapeptide	Q-Exactive-HF	DDA	Comet	6918 peptides
Xiao et al.^[52]^	2018	synthetic microbial community; fecal sample	MT	LTQ-Orbitrap	DDA	X! Tandem MaxQuant	11308 peptides; 10932 peptides
Long et al.^[20]^	2020	fecal sample	hybrid spectra library-based DIA	Orbitrap Fusion Lumos Tribrid	DIA	PEAKS SpectroMine Spectronaut	91902 peptides; 30062 proteins
Aakko et al.^[21]^	2020	synthetic microbial community; fecal sample	DDA spectra library-based DIA	Q Exactive HF	DIA	diatools	14888 peptides; 12804 peptides
Stamboulian et al.^[53]^	2021	human colonoscopy sample	HAPiID	LTQ-Orbitrap	DDA	MS-GF+	24962 peptides
Pietilä et al.^[54]^	2022	fecal sample	directDIA	Q Exactive HF	DIA	X!Tandem Comet	14691 peptides
Zhao et al.^[55]^	2023	synthetic microbial community; fecal sample	directDIA	Orbitrap Fusion Lumos Tribrid	DIA	PEAKS MaxQuant FragPipe	10572 proteins; about 70000 proteins
Gómez-Varela et al.^[47]^	2023	mice fecal sample	peptide-centric DIA analysis	timsTOF Pro	DIA	DIA-NN MaxQuant	over 15000 proteins
Wu et al.^[56]^	2024	synthetic microbial community; fecal sample	ConDiGA	timsTOF Pro	DDA	PEAKS	13537 proteins; 12630 proteins
Wu et al.^[57]^	2024	synthetic microbial community; fecal sample	HAPs-hyblibDIA	timsTOF Pro	DIA	Spectronaut	11770 proteins; 28585 proteins

MetaPro-IQ: metaproteome identification and quantification; Metapeptide: short amino acid sequences that may be represented in multiple organisms; MT: metagenomic taxonomy-guided database-search strategy; HAPiID: high abundance proteins guided metaproteomics identification; ConDiGA: contigs directed gene annotation; HAPs-hyblibDIA: high-abundance protein-guided hybrid spectral library for data-independent acquisition metaproteomics.

## 3 质谱数据分析

### 3.1 DDA数据分析策略

#### 3.1.1 数据库搜索

宏蛋白质组学DDA数据分析包含数据库搜索和*de novo* sequencing两种方法。数据库搜索的基本流程是首先将蛋白质序列数据库进行理论酶切产生肽段,生成理论二级谱图;将理论二级谱图与实验二级谱图进行匹配,按照一定规则打分得到肽段-谱图匹配结果(peptide-spectrum match, PSM);对搜库结果进行质控,得到最佳匹配的肽段,然后进行蛋白质推断得到蛋白质鉴定结果。数据库搜库是宏蛋白质组学DDA分析中最常用的分析策略。在数据库搜库的解析方法中,首先要构建合适的数据库。数据库的大小和完整性不仅对鉴定数量有很大影响,还会影响物种和功能水平上的分析。理想情况下,数据库应该包含样本中存在的所有物种的蛋白质序列,且不包含多余序列。如图4所示,目前的蛋白质序列数据库可分为三类:公共数据库或基于公共数据库优化的数据库、基于宏基因组测序数据得到的数据库和基于16/18S rRNA测序的伪宏基因组数据库。常用的公共数据库有NCBI非冗余细菌数据库(http://www.ncbi.nlm.nih.gov/)、UniProt非冗余细菌库(https://www.uniprot.org/)、肠道微生物蛋白质数据库IGC (https://db.cngb.org/microbiome/genecatalog/genecatalog_human/)、人类微生物组计划产生的蛋白质数据库HMP (https://www.hmpdacc.org/)和人类胃肠道微生物蛋白质数据库UHGP(https://bcb.unl.edu/dbpup/uhgp_home)等。然而,使用大型公共数据库不可避免地会导致搜索空间膨胀,搜库时间增加,鉴定灵敏度降低。另一方面,过大的数据库往往包含过多的冗余序列,增加序列相似性,使蛋白质推断变得更加复杂。

**图4:**
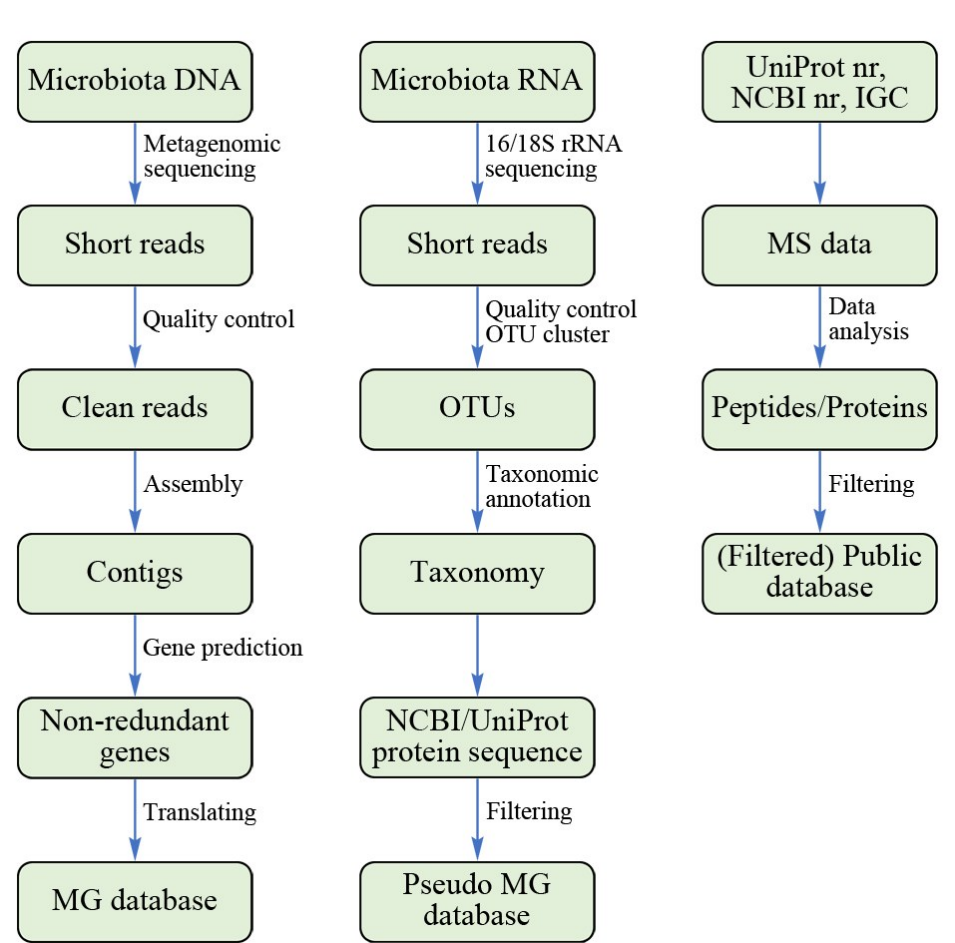
蛋白质序列数据库构建方式

从同一样本的宏基因组测序数据得到蛋白质序列数据库称之为宏基因组匹配的蛋白质数据库,其只包含样本中存在的蛋白质序列,具有较小的搜索空间。基于二代测序的宏基因组数据库构建流程是首先对微生物群落样本进行鸟枪法宏基因组测序得到短读序列(short reads),将short reads组装成重叠群(contigs),然后进行基因预测、注释和翻译,最后得到该样本的蛋白质序列数据库。基于宏基因组的蛋白质序列数据库与样本实际存在的蛋白质序列最为接近,并且具有较小的搜索空间,是目前宏蛋白质组学中数据库构建的金标准。尤其是对于大多数组成未知或者相关研究较少的微生物群落样本,宏基因组测序得到的蛋白质序列数据库是最佳选择。但是,基于宏基因组的蛋白质序列数据库也可能存在测序深度不足、测序错误、组装错误、注释错误等问题。

另一种数据库构建方式是基于16S rRNA扩增子测序数据的分类信息来过滤UniProt等公共数据库,也被称为伪宏基因组蛋白质序列数据库。然而,16S rRNA测序在种甚至属层级的分辨率有限,使得该数据库存在诸多问题。如果在属层级构建数据库,会包含样品中不存在的大量蛋白质序列,增加计算时间,降低检测灵敏度。而在物种层级构建的数据库可能会遗漏样品中存在的蛋白质序列信息。此外,公共数据库中收录的物种还很不完整,许多已知物种没有完整的蛋白质组,大量未知物种还没有被测序。

针对数据库规模过大引起的分析问题,研究人员开发了一系列优化策略。其中最常用的是迭代搜库策略,迭代搜库策略的中心思想是通过多次搜库来缩小序列数据库规模。Jagtap等^[58]^提出了两步迭代搜库方法,在第一次搜库时不设置任何过滤参数,保留所有的蛋白质鉴定结果,用于构建一个缩小的数据库进行第二次常规搜库,此时再设置严格的错误发现率(false discovery rate, FDR)过滤参数得到最终鉴定结果。Zhang等^[59]^提出了三步迭代搜库方法,并构建了宏蛋白质组分析流程MetaPro-IQ,该方法在两次迭代搜库结果的基础上,进行第三次常规搜库以得到最终鉴定结果。迭代搜库策略可以有效缩小数据库大小,从而提高蛋白质鉴定数量。然而,第一步直接搜索大的数据库仍然非常耗时,Stamboulian等^[60]^提出了HAPiID方法,他们收集了4个数据库HMP、UMGS (Uncharaterised MetaGenome Species)^[61]^、HBC (Human Gastrointestinal Bacteria Culture Collection)^[62]^以及文献报道的古菌(Archea)^[63]^,合并为更加全面的人肠道微生物宏蛋白质组数据库,然后提取该数据库中的高丰度蛋白质即核糖体蛋白质和延长因子,构建了一个高丰度蛋白质数据库。使用该高丰度蛋白数据库进行第一轮搜库,根据搜库结果推断出样本中包含的物种,然后构建一个包含这些物种蛋白质组的数据库。应用该方法对人体肠道微生物进行宏蛋白质组分析,不仅极大地减少了搜库时间,同时提高了蛋白鉴定数量。

#### 3.1.2 *de novo* sequencing策略

与数据库搜索不同的是,*de novo* sequencing策略不依赖于序列数据库进行比对分析,而是直接通过MS^2^谱图中碎片峰的*m/z*差异来推导出肽段序列。该方法能够发现未知序列或具有变异的蛋白质序列,但是由于MS^2^谱图中碎片峰信息很可能不完全,因此会导致蛋白质鉴定数量较少并且准确性不足。因此有研究者首先基于*de novo* sequencing策略获得短的序列标签,再利用该序列标签进行数据库搜索^[64]^。2018年,Lee等^[19]^在预印本上发表的研究中首次将*de novo* sequencing方法应用于宏蛋白质组分析中(该文章于2022年最终发表),该研究建立了新的谱图注释工具Kaiko,成功地从宏蛋白质组数据中鉴定出单一菌株和合成微生物群落的物种组成。作者利用Kaiko获得的物种构建了不依赖于宏基因组测序的数据库,并将其应用于真实土壤样本的宏蛋白质组分析。该方法成功鉴定出16S rRNA测序得到的所有高丰度物种,并发现了一些仅在蛋白质组学数据中鉴定到的物种,对这些蛋白质进行功能分析得到1059种酶,其中618种酶被映射到KEGG(Kyoto Encyclopedia of Genes and Genomes)代谢通路中。作者依据这些映射绘制了土壤微生物群落的代谢通路图谱。2021年,Kleikamp等^[18]^建立了基于*de novo* sequencing的宏蛋白质组分析流程NovoBridge,利用合成微生物群落和自然微生物群落证明了该方法对微生物群落的分类组成分析能力和定量分析能力。2023年,Potgieter等^[65]^开发了一种基于*de novo* sequencing的数据分析方法MetaNovo,该方法利用*de novo*序列标签信息对Uniprot全库进行过滤来构建数据库,再进行数据库搜索。

### 3.2 DIA数据分析策略

DIA数据中包含着全面的碎片离子信息,每张MS^2^谱图包含多种母离子的碎片离子峰,因此数据解析难度大。尤其是对于宏蛋白质组来说,DIA数据更加复杂,其数据解析面临更大的挑战。宏蛋白质组DIA数据分析的策略主要分为以肽段为中心(peptide-centric)和以谱图为中心(spectrum-centric)两类。

典型的以肽段为中心的策略,通常需要利用同一样本的DDA数据预先构建谱图库,再利用该谱图库对DIA数据进行解析。该谱图库的构建过程与常规DDA搜库过程类似,需要将DDA谱图与蛋白质序列数据库进行比对得到DDA的鉴定结果。2020年,Aakko等^[66]^利用DDA数据构建谱图库解析宏蛋白质组DIA数据,首次将DIA应用在宏蛋白质组分析中。然而这种方法使DIA的鉴定和定量结果受限于DDA的鉴定结果。随着搜库软件算法的改进,可以通过结合DDA数据和DIA数据的搜库结果构建一个混合谱图库(hybrid spectral library),利用混合谱图库解析DIA数据可以提高DIA的覆盖深度。Long等^[9]^用SpectroMine对结直肠癌(colorectal cancer, CRC)队列样本的DDA数据和DIA数据搜库分析生成了一个混合谱图库,从28份临床粪便样本中鉴定到91902个肽段和30062个蛋白质组。通过对CRC患者和健康对照组肠道菌群的差异蛋白质进行功能分析,揭示了肠道菌群中的铁摄取与转运和氧化应激过程在结直肠癌发病及发展过程中扮演的角色。此外,通过深度学习算法预测工具,如DeepDIA^[67]^、Prosit^[68]^和DIANN^[69]^等,可以从蛋白质序列数据库直接生成预测谱图库,再进行DIA数据解析。但是目前该方法还缺少在宏蛋白质组中的应用。

以谱图为中心的策略,也被称为directDIA,可以直接对DIA数据进行分析而不需要预先构建谱图库。其原理是基于母离子和碎片离子的色谱流出相似性,从DIA数据中生成每个母离子的伪二级谱图,然后进行类似于DDA数据库搜索构建内部谱图库或者伪谱图库,再解析DIA数据。2022年,Pietilä等^[54]^首次利用该方法对复杂的宏蛋白质组样本DIA数据直接分析,克服了基于DDA建库的局限性。2023年,Zhao等^[70]^应用该方法(directDIA)对肠道微生物样本进行宏蛋白质组学研究,并比较了不同方法的定量结果。结果表明,directDIA在鉴定和定量准确性方面表现优异,证明了directDIA在肠道微生物样本中进行宏蛋白质组定量研究的可行性。

在DIA数据分析中,蛋白质序列数据库构建同样对最终鉴定结果有着重要的影响。但由于DIA包含了更加复杂的二级谱图信息,使用大数据库需要更长的搜库时间和计算资源。最近,Wu等^[71]^开发了一种基于高丰度蛋白质(high abundance proteins, HAPs)的宏蛋白质组DIA分析策略。首先从UniProt数据库中下载了647种肠道微生物的核糖体蛋白质和延长因子,构建了一个肠道微生物群HAPs数据库。然后将UMGS数据库中的HAPs与这647个物种的HAPs进一步结合,建立了一个综合的HAPs数据库。通过使用该HAPs数据库搜索DIA数据,确定样本中包含的物种。基于该物种信息构建样本特定的全蛋白质组数据库,对DIA数据进行分析。结果表明,该方法可以准确地鉴定微生物群样品的分类组成并实现高效、高灵敏宏蛋白质组分析。

## 4 物种分类和功能注释

微生物群落在不同分类学层级的组成是微生物组的研究重点之一。近年来开发了一系列注释工具,可以在蛋白质水平、肽段水平、基因水平上进行物种注释来获得微生物群落组成。在蛋白质水平上的物种注释主要是基于序列相似性进行比对,注释工具包括BLAST^[72]^和GhostKOALA(https://www.kegg.jp/ghostkoala/)等。在肽段水平进行物种注释一般使用Unipept(https://unipept.ugent.be/)注释工具,其利用NCBI数据库以及UniProt数据库和最近公共祖先算法(LCA)对宏蛋白质组鉴定到的肽段进行物种的注释。但是由于其数据库涵盖范围非常广,在对肽段序列进行搜索比对时,物种水平的注释率较低。基于宏基因组构建的蛋白质数据库作为宏蛋白质组数据库的金标准,其物种注释结果指导了宏蛋白质组的物种注释。宏基因组学开发了一系列注释工具,如Kraken2^[73]^、BLAST^[72]^、Kaiju^[74]^和MEGAN^[75]^等,可以对宏基因组测序数据在reads、contigs、genes等层面进行分类注释。最近,我们开发了一种新的物种注释策略ConDiGA(contigs directed gene annotation)^[56]^。在该策略中,首先对contigs进行物种注释,然后基于基因组覆盖率和分类丰度从contigs的注释结果中选取候选物种,利用这些物种的参考基因组来构建一个基因序列数据库,最后基于这个基因序列数据库,对预测的基因进行物种水平的注释。利用ConDiGA策略,在合成微生物群落样本中实现了100%的注释准确度,在粪便样本中鉴定到的蛋白质注释率达90%以上,相比主流方法提升100%以上。

功能注释的本质是将目标蛋白质序列与功能蛋白质序列数据库进行比对。利用基因功能数据库如GO (https://geneontology.org/)、COG (http://www.ncbi.nlm.nih.gov/COG/)、KEGG (https://www.genome.jp/kegg/)、eggNOG (http://eggnogdb.embl.de/)等,可以对宏蛋白质组鉴定到的蛋白质进行不同的功能注释分析,常用的注释工具有Blast2GO^[76]^、DAVID^[77]^、KOBAS^[78]^等。与其他组学方法相比,微生物群落的功能分析是宏蛋白质组学的一个独特特征。目前,宏蛋白质组以外的微生物组学研究方法主要有宏基因组和宏转录组。通过宏基因组分析,可以确定一个环境样本中存在哪些微生物,并揭示这些微生物可能具备哪些代谢途径和生物功能。然而,宏基因组数据只能提示某一环境中可能存在的功能基因,却不能反映这些基因是否被表达或在特定条件下的表达水平。宏转录组学关注的是在特定时间点在特定环境条件下,微生物群落中所有mRNA组成。通过宏转录组学分析,可以了解在特定环境条件下,哪些基因处于活跃状态,从而预测哪些代谢途径在群落中被激活。尽管宏转录组学提供了关于基因表达的上游信息,但RNA水平的变化并不总是直接对应蛋白质活动的改变。

目前,基于宏蛋白质组的功能分析已经成为微生物研究领域的一大热点。相较于宏基因组学和宏转录组学,宏蛋白质组学直接研究微生物群落中的蛋白质,而蛋白质是生物学功能的直接执行者。通过宏蛋白质组学分析,研究人员可以定量分析微生物群落中的蛋白质表达及其变化,从而识别出涉及病原体、抗性发展、代谢途径等特定生物学功能的蛋白质。通过分析微生物信号转导通路相关的蛋白质,可深入理解微生物与宿主之间的相互作用机制。将蛋白质表达数据与微生物群落的结构数据结合起来,可以分析特定群落结构与生物学功能之间的关系。除此以外,蛋白质组学不仅可以分析蛋白质的表达水平,还可以研究蛋白质的后修饰状态和蛋白质之间的相互作用,这些都是研究生物学功能调控的重要信息。Jouffret等^[79]^对土壤样本的宏蛋白质组和宏基因组的功能分析表明,宏蛋白质组可以更好地检测到微生物群落中信号转导与互作相关的基因表达。这一结果表明,从基因层面的功能分析难以真实反映微生物群落表达的功能,而利用宏蛋白质组可以更好地研究微生物群落表达的蛋白质、激活的代谢通路和发挥的功能。因此,与宏基因组学和宏转录组学相比,宏蛋白质组学在揭示微生物群落的实际功能方面具有独特的优势,能够直接体现出在特定条件下微生物群落的功能状态和生物学活动,为理解微生物在环境和宿主中的作用提供了重要的视角。

## 5 总结与展望

微生物在人类健康和疾病中扮演着重要角色。近年来,宏蛋白质组学已经成为研究微生物群落功能的重要技术手段。宏蛋白质组学的分析流程与单一物种蛋白质组类似,但是由于宏蛋白质组研究对象的复杂性,从样品前处理、数据采集到数据分析等各个分析步骤中需要采用特定的研究策略。目前,得益于前处理方法的改进、质谱技术的不断革新和生物信息学的快速发展,宏蛋白质组学在鉴定深度、应用范围等方面取得了长足的进展。

在宏蛋白质组样品前处理过程中,首先需要考虑样本的性质,如何将微生物与环境细胞和蛋白质分离是宏蛋白质组面临的关键挑战之一,分离效率与微生物损失的平衡是亟待解决的问题。其次,微生物的蛋白质提取要考虑到不同细菌的结构异质性带来的差异,宏蛋白质组样品处于微量范围也需要特异的前处理方法。

质谱仪器方面,主流的质谱仪器经历了从LTQ-Orbitrap、Q Exactive等基于Orbitrap质量分析器的质谱仪到timsTOF Pro等基于离子淌度耦合飞行时间质量分析器的质谱仪的转变。具有离子淌度维度信息的timsTOF系列仪器检测准确度高、检出限低、重复性好,已经逐渐在单一物种的蛋白质组、宏蛋白质组、代谢组等多种需要质谱检测的研究领域成为重要仪器。值得注意的是,长期以来,质谱仪器的动态范围限制了宏蛋白质组研究的蛋白质覆盖深度,未来具有更大的动态范围的质谱仪器可以提高宏蛋白质组蛋白质鉴定的灵敏度与准确性。

对于质谱数据采集,尽管在单一物种的蛋白质组中,DIA数据采集模式已经被广泛采用,但是当前大多数宏蛋白质组分析仍然采用的是DDA数据采集模式。DIA数据采集模式能完整地获取样品的碎片离子信息,相较DDA数据采集模式有完整获取宏蛋白质组样品肽段信息的潜力,但是由于DIA数据的高度复杂性,目前DIA宏蛋白质组数据的解析仍然面临着极大的困难,人工智能和深度学习的发展有望提高DIA数据解析的准确度与完整性。

在宏蛋白质组的数据分析中,关键步骤之一在于蛋白质序列数据库的构建。对于热门研究领域如肠道菌群,可以采用IGC和HMP等肠道微生物数据库,并已取得很好的鉴定结果。对于其他大多数的宏蛋白质组学分析,最有效的数据库构建策略仍然是基于宏基因组测序数据建立样本特异的蛋白质序列数据库。对于复杂性高、动态范围大的微生物群落样本,需要增加测序深度,以增加对低丰度物种的鉴定,从而提高蛋白质序列数据库的覆盖度。当缺乏测序数据时,可以采用迭代搜索方法优化公共数据库。然而,迭代搜索可能会影响FDR质控,因此需要对搜库结果进行仔细检查。此外,对于传统的FDR质控模型在宏蛋白质组分析中的适用性仍然值得探索。在搜索策略方面,混合谱图库策略可以提高DIA宏蛋白质组学的覆盖深度。近几年,基于深度学习生成的预测谱图库在DIA蛋白质组学中表现出了优越的性能。然而,宏蛋白质组数据库往往包含上百万个蛋白质条目,这会导致预测的谱图库规模过大,需要消耗极大的计算资源,并且会导致搜索空间过大。除此以外,宏蛋白质组的蛋白质序列间相似度差异大,难以确保谱图库预测模型的准确度,因此预测谱图库还没有在宏蛋白质组学中被广泛应用。此外,还需要开发新的蛋白质推断和分类注释策略,以适用高度序列相似的宏蛋白质组学分析。

综上所述,作为一门新兴的微生物组研究技术,宏蛋白质组技术在已经取得重大研究成果的同时,也保有着巨大的发展潜力。
